# Prediction of Chromatographic Elution Order of Analytical Mixtures Based on Quantitative Structure-Retention Relationships and Multi-Objective Optimization

**DOI:** 10.3390/molecules25133085

**Published:** 2020-07-06

**Authors:** Petar Žuvela, J. Jay Liu, Ming Wah Wong, Tomasz Bączek

**Affiliations:** 1 Department of Chemistry, National University of Singapore, Singapore 117543, Singapore; petar.zuvela@nus.edu.sg (P.Ž.); chmwmw@nus.edu.sg (M.W.W.); 2 Department of Chemical Engineering, Pukyong National University, Busan 48513, Korea; 3 Department of Pharmaceutical Chemistry, Medical University of Gdańsk, 80-416 Gdańsk, Poland; tbaczek@gumed.edu.pl

**Keywords:** reversed-phase high performance liquid chromatography, elution order prediction, quantitative structure-retention relationships, multi-objective optimization

## Abstract

Prediction of the retention time from the molecular structure using quantitative structure-retention relationships is a powerful tool for the development of methods in reversed-phase HPLC. However, its fundamental limitation lies in the fact that low error in the prediction of the retention time does not necessarily guarantee a prediction of the elution order. Here, we propose a new method for the prediction of the elution order from quantitative structure-retention relationships using multi-objective optimization. Two case studies were evaluated: (i) separation of organic molecules in a Supelcosil LC-18 column, and (ii) separation of peptides in seven columns under varying conditions. Results have shown that, when compared to predictions based on the conventional model, the relative root mean square error of the elution order decreases by 48.84%, while the relative root mean square error of the retention time increases by 4.22% on average across both case studies. The predictive ability in terms of both retention time and elution order and the corresponding applicability domains were defined. The models were deemed stable and robust with few to no structural outliers.

## 1. Introduction

High-performance liquid chromatography in the reverse-phase separation mode (RP-HPLC), accounts for more than 90% of separations in modern analytical laboratories [1]. Prediction of LC retention time has become valuable, powerful, and routine in chromatographic method development. Depending on the experimental design, researchers may be directly interested in retention data, or may use them to infer additional information. The quantitative structure-retention relationships (QSRR) model provides significant additional insight into the relationship between the molecular structure and fundamental phenomena in chemistry.

QSRRs can be useful in a variety of applications, such as identification of the most useful structural descriptors that describe the retention mechanism, prediction of retention time of new analytes, and the identification of unknown analytes. It can also be used for the quantitative comparison of separation properties of different chromatographic columns; for evaluation of physical properties, such as lipophilicity or dissociation constants; as well as estimation of relative bioactivities of xenobiotics [2].

**Figure 1:**
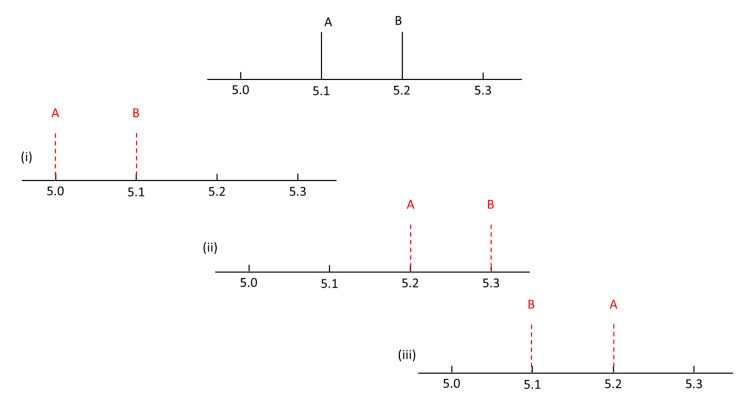
Schematic depiction of three chromatograms with identical retention time error, but different elution order errors (vertical solid lines in black: true retention time of analyte A and B; vertical dotted lines in red: predicted retention time of analyte A and B).

An applicable retention time prediction model not only needs to be predictive, but also needs to be able to predict analytes in the correct order. Suppose one has two close chromatographic peaks 0.1 min apart, as schematically depicted in Figure 1. For instance, 5.1 min for analyte A, and 5.2 min for analyte B. The following three cases yield identical retention time errors, but different elution order errors: predicted retention times: (i) *t*_R,A_ = 5.0 min, *t*_R,B_ = 5.1 min; (ii) *t*_R,A_ = 5.2 min, *t*_R,B_ = 5.3 min; and (iii) *t*_R,A_ = 5.2 min, *t*_R,B_ = 5.1 min. In the aforementioned example, cases (i) and (ii) still remain satisfactory as the order has not shifted. The third case, however, involves a shift in the elution order despite the identical error in retention order. Such a simple example already emphasizes the problems that this work aims to solve.

This becomes especially evident in the case of very complex mixtures with hundreds of compounds (such as peptides in a proteomic mixture) whereby the peak capacity greatly increases. In RP-HPLC, solutes are eluted in order of decreasing polarity. However, it is often not as straightforward, and a retention time prediction model utilizing hydrophobicity/lipophilicity as a predictor is often insufficient to provide a clear picture of the retention mechanism. Thereby, even though the model may predict the retention time with a reasonable error, the predicted order of analytes for complex analytical mixtures containing thousands of close or even overlapping peaks is often very poor. Approaches to solve this complex chromatographic problem in RP-LC are quite rare, and outlined in a few studies in the literature [3,4,5,6]. In a QSRR study involving solvatic sorption parameters [7] for prediction of retention of phenylisothiocyanate derivatives of 25 natural amino acids, Vorslova et al. [3] report a retention error of <6%. While the predicted elution order mostly concurred with the experimental one, for retention times >15 min the QSRR model yielded larger deviations, with a few unresolved peaks.

Another example of elution order prediction is in a work of Shinoda et al [4]. The authors utilized artificial neural networks (ANNs) to predict retention times of peptides (<50 amino acids) and report a reasonable model for 834 peptides (with *R*^2^ of 0.928). Although not the first time the use of ANNs have been reported in retention time prediction in proteomics [8,9], Shinoda et al. [4] are the first to report the prediction of the elution order with an error of elution order <11%. The method itself does not integrate the elution order prediction directly into the modelling process and it is rather vaguely described.

Recently, a complex methodology was presented by Bach et al. [5] for elution order prediction in metabolomics using support vector machines [10] and dynamic programming [11]. The approach was based on molecular fingerprints of two molecules as the input and the elution order as the output. Although quite promising, the method is computationally expensive, whereas the predictions of the elution order are sensitive to the number of training samples and their composition [5].

In this work, we define the elution order prediction as a multi-objective optimization problem with two objective functions: percentage root mean square error (%RMSE) of retention time and that of elution order. It is directly implemented within the QSRR modelling process, where regression coefficients are determined through multi-objective optimization (MOO) employing the two objectives, root mean square errors of retention time and elution order. MOO was used to obtain QSRR models which compromise prediction errors in favor of the enhanced elution order prediction. The developed method was applied to analysis of two mixtures: (i) separation of organic molecules on Supelcosil LC-18 column, and (ii) separation of peptides in seven columns under varying chromatographic conditions.

## 2. Results and Discussion

This work presents a method for the prediction of the elution order in RP-LC based on QSRRs defined as an MOO problem. Two case studies have been evaluated in eight RP-LC columns and under various experimental conditions. Since the current study represents a proof-of-concept for preliminary evaluation of our method, data for two case studies were taken from two literature sources. Namely, the works of Kaliszan et al. [12] and Bączek et al [13]. Case study 1 (CS1) was represented by a single chromatographic column (Supelcosil LC-18). Case study 2 (CS2) on the other hand was represented with multiple columns and gradients. Inclusion of all the chromatographic columns into our conceptual work was to exhibit the versatility of the approach and to emphasize the differences in elution order predictability.

**Figure 2:**
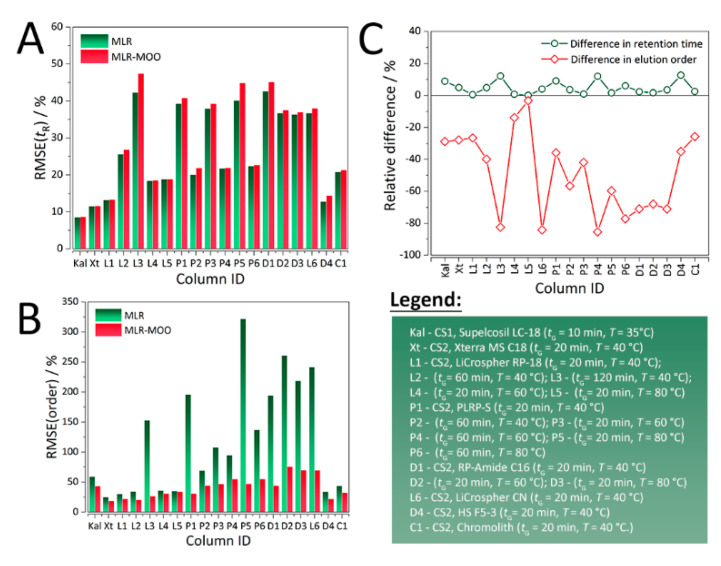
Distribution of %RMSE values of MLR (control) and MLR-MOO models for (**A**) retention time, (**B**) elution order, and (**C**) relative difference in retention time and elution order RMSE values between MLR and MLR-MOO. In (**A**,**B**) the color bars are in sequence (first MLR, then MLR-MOO). In (**C**) the lines with open circle shapes represent the relative differences in retention time, whereas the lines with open diamond shapes represent the differences in elution order. All the abbreviations are explained in the text.

In both case studies, for all the RP-LC columns, sacrificing retention time predictive ability (Figure 2A) resulted in a considerable increase in prediction of elution order (Figure 2B). As evident from Figure 2C and Table 1, the maximum relative increase in %RMSE(*t*_R_) is a little over 15%, whereas there is up to > 80% decrease in %RMSE(order). The molecular descriptors, experimental and predicted retention times for both MLR and MLR-MOO are summarized in Tables S2 and S3, respectively.

Table 2 summarizes the physicochemical parameters of the investigated columns. Namely, column length, internal diameter, particle size, carbon load, pore size, and surface area. When it comes to predictability of selectivity, and by extension the elution order, column length and internal diameter are very important parameters. When the dwell volume is high, a low flow rate makes it more difficult to predict elution order, because of delayed gradient elution. Larger internal diameter leads to higher flow rates, whereas a decrease in the internal diameter is favorable for faster analyses but results in an increase in back pressure which may also adversely affect the predictability of the elution order. Column length positively affects analysis time, while decreasing resolution. For higher column lengths, elution order may be more predictable. However, elution order predictability is more crucial to columns with lower lengths due to decreased peak spacing.

From Table 2 it can be observed that most of the columns have the same internal diameter (4.6 mm) except PLRP-S with a value of 4.1 mm. On the other hand, all the columns are reasonably long (>150–200 mm), apart from Chromolith and LiCrospher CN which have a length of 100, and 125 mm, respectively. For LiCrospher CN, this fact is reflected in considerably higher error in elution order for the control MLR model (%RMSE(order) = 195.82%). Columns with other lengths exhibit various %RMSE(order) values, with some as high as 241.63% for Discovery RP Amide C16 with a 20-min gradient and a temperature of 80 °C. These kinds of inconsistencies may have occurred because of the missing dwell volume information not reported in the works of Kaliszan et al. [12] and Bączek et al. [13] from which the retention data were obtained. In QSRR modelling, physicochemical parameters of the column and the column itself are kept constant and it is assumed that retention depends mostly on the molecular structure of the analytes [14,15]. Lack of generalizability and dependence on a pre-defined set of chromatographic conditions is not only the main limitation of QSRR, but of QSAR and QSPR. However, such dependence diminishes the influence of physicochemical parameters of the column on prediction of retention time and order.

**Figure 3:**
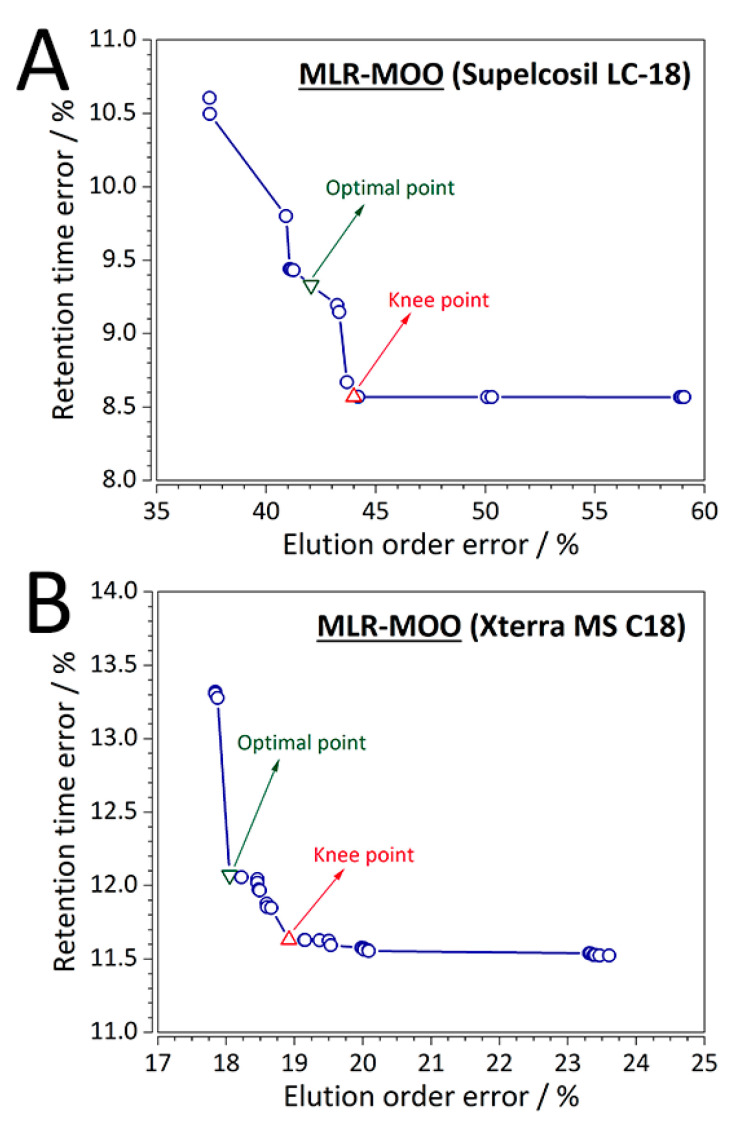
MLR-MOO Pareto front for the (**A**) first case study (Supelcosil LC-18 column, *t*_G_ = 10 min, *T* = 35 °C), and for (**B**) the second case study (Xterra MS C18 column, *t*_G_ = 20 min, *T* = 40 °C).

Out of the eight RP-LC columns evaluated, results of two representative columns for each case study (first case study—CS1: Supelcosil LC-18, *t*_G_ = 10 min, *T* = 35 °C; and second case study—CS2: Xterra MS C18, *t*_G_ = 20 min, *T* = 40 °C) are summarized in more detail as an example. For the MLR-MOO models, the optimal solution (solution 19, %RMSE(*t*_R_) = 8.670%, %RMSE(order) = 43.679%) for CS1 (Supelcosil LC-18 column) is shown in Figure 3A, while the optimal solution (solution 16, %RMSE(*t*_R_) = 11.631%, %RMSE(order) = 19.820%) for one of the representative columns of the second case study (Xterra MS C18 column) is shown in Figure 3B. As mentioned in one of the subsequent sections (Section 3.7), it is up to the user to set an upper bound on the loss of retention predictive ability. For instance, for CS1 involving the Supelcosil LC-18 column the limit was set to ~10% from the knee point value. For the Supelcosil LC-18 column the increase in %RMSE(*t*_R_) was 8.67, whereas the decrease in %RMSE(order) was 4.43%. One may argue that the point with the lowest %RMSE(*t*_R_) is the optimal one (Figure 3A). However, if indeed deemed optimal, the point decreases the %RMSE(order) by 14.94, while at the same time increases the %RMSE(*t*_R_) by a sizeable 19.23%. Similarly, the MOO Pareto front was analyzed for the Xterra MS C-18 (Figure 3B) column of CS1 where the limit was set to ~5%. Consequently, for an increase of %RMSE(*t*_R_) by 3.65, a decrease in the %RMSE(order) of 4.82% was achieved.

We reiterate that setting an upper bound on the increase of %RMSE(*t*_R_) is a user-defined parameter, and in such a case there is “no free lunch” [16].

**Figure 4:**
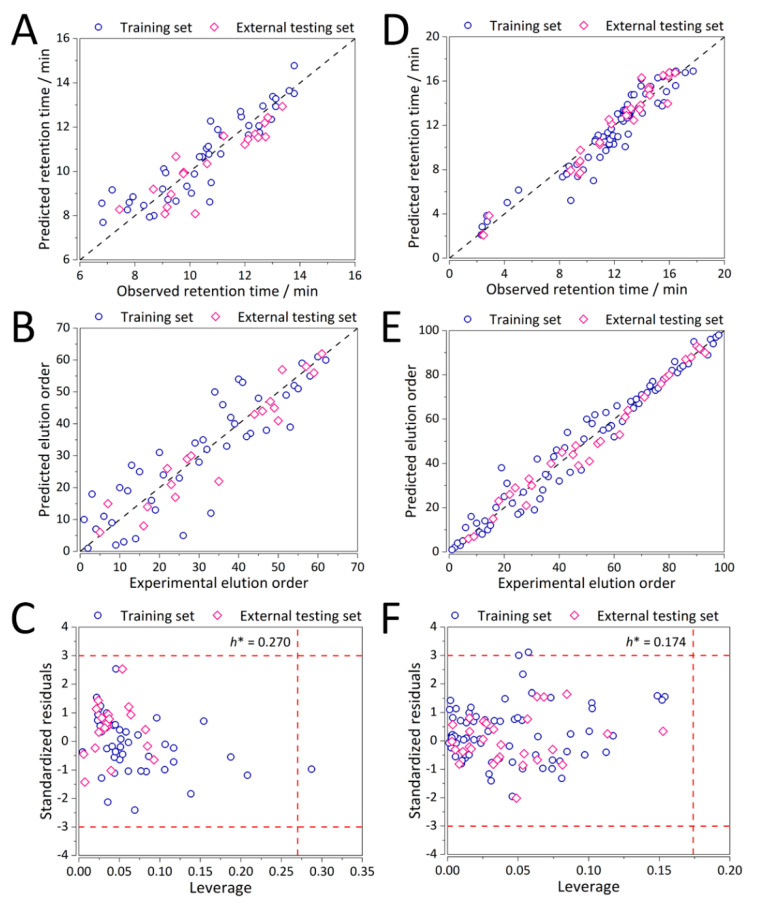
Performance of the MLR-MOO method in prediction of (**A**) retention time, (**B**) elution order, and (**C**) applicability domain for CS1 (Supelcosil LC-18, *t*_G_ = 10 min, *T* = 35 °C), (**D**) prediction of retention time, (**E**) elution order, and (**F**) applicability domain for CS2 (Xterra MS C18, *t*_G_ = 20 min, *T* = 40 °C). Blue open circle shapes represent the training set, whereas the pink open diamond shapes represent the testing set observations.

Predictive ability in terms of both retention time and elution order and the corresponding (analytical chemical) domains of applicability for the optimal MLR(-MOO) QSRR models for the representative columns of CS1 and CS2 for MLR(control) and MLR-MOO calculations are depicted in Figure S1 and Figure 4, respectively. Reasonable performance is exhibited both for CS1 (Figure 4A–C), and CS2 (Figure 4D–F).

**Figure 5:**
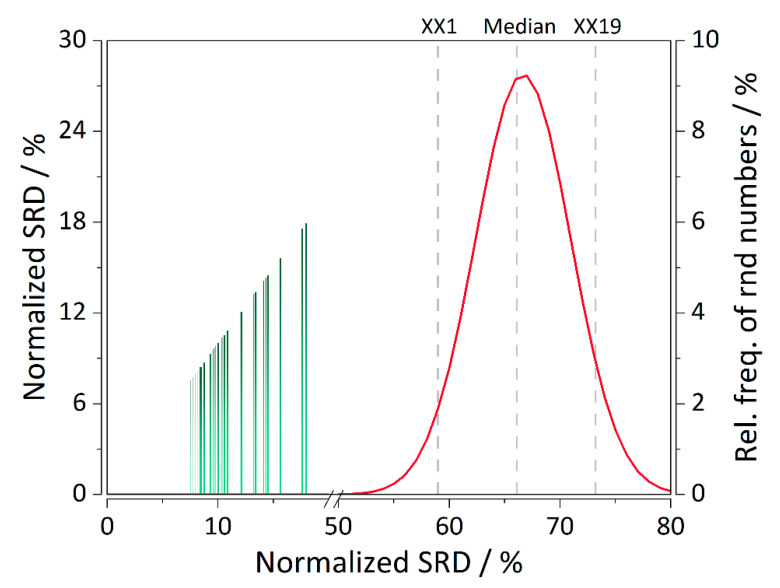
SRD analysis ranking the columns of the second case study according to their respective elution order predictions (“standard”: experimental elution order). Validated with normally distributed random numbers. SRD value of the first icosaile (XX1) was 59.00, median 66.10, whereas the last icosaile (XX9) was 73.2. Barplot legend (sorted ascending by SRD values): Xt(MLR-MOO) = 7.58; L2(MLR-MOO) = 7.71; L1(MLR) = 7.79; L4(MLR-MOO) = 7.91; L1(MLR-MOO) = 8.00; Xt(MLR) = 8.12; C1(MLR-MOO) = 8.21; L5(MLR) = 8.33; L4(MLR) = 8.46; L5(MLR-MOO) = 8.75; C1(MLR) = 9.25; D4(MLR-MOO) = 9.33; P4(MLR-MOO) = 9.54; L3(MLR-MOO) = 9.58; P6(MLR-MOO) = 9.70429; P2(MLR-MOO) = 9.82; P1(MLR), P3(MLR-MOO) = 9.96; L2(MLR) = 10.04; L6(MLR-MOO) = 10.37; P1(MLR-MOO) = 10.41; D4(MLR) = 10.58; P3(MLR) = 10.83; P5(MLR-MOO) = 10.87; P5(MLR) = 12.12; L6(MLR) = 13.20; P4(MLR) = 13.25; D3(MLR-MOO) = 13.29; P2(MLR) = 13.42; L3(MLR) = 14.16; P6(MLR) = 14.37; D3(MLR) = 14.54; D1(MLR-MOO) = 15.62; D2(MLR-MOO) = 15.66; D2(MLR) = 17.62; D1(MLR) = 17.95. Readers are kindly referred to Figure 2 for the definitions of the abbreviations. SRD values summarized in Table 2.

Nearly all the analytes within both case studies are within their respective applicability domains (Figure 4C,F) with only one structurally important analyte which is predicted well and was included in the training set. Thereby, the developed models are deemed stable and robust to structurally more distant analytes. Performance of all the columns in CS2 involving the separation of synthetic peptides in terms of elution order predictions was ranked using SRD analysis. Figure 5 depicts the ranking values for all the columns in CS2. Validation was performed by computing SRD values for normally distributed random numbers. It can be observed that the ranking of all the QSRR models of the second study is statistically significant because their respective SRD values are on the far-right side of the Gaussian curve. A few columns stand out, especially the Discovery RP Amide C16, in all the combinations of gradients and column temperatures, which exhibited that errors in the elution order are up to 70% even after MOO. It is then not surprising that they are ranked among the last. Even in the original work of Bączek et al. [13] from which the data have been obtained the models in question performed poorly. It is worth noting that these columns are highly polar and their respective polar intramolecular interactions with the analytes are difficult to quantify [13]. Therefore, the current QSRR model was not able to capture the retention mechanism in its entirety. Nevertheless, our method has still decreased the %RMSE(order) approx. three-fold, from ~200% to ~70%.

As for the other columns of the second case study, there are a few interesting examples. For instance, for the PLRP-S column, %RMSE(*t*_R_) systematically increases as the gradient decreases from 60 to 20. On the other hand, for the LiChrospher RP-18 column it is the opposite. %RMSE(*t*_R_) for this column increases with the increase of the total gradient time. The increases in the errors may be due to the absence of isomeric peptides, so the QSRR models have not accounted for the proximity effects of two or more identical amino acids in the peptides’ primary sequence. Retention of the peptides, such as the “AA” peptide, do not necessarily correlate strongly with a simple sum of their respective gradient time “basis sets”. The linear model applied in this work may not be sufficient to account for this behavior. In both cases the analytes exhibiting lower retention times exhibit a degree of non-linearity between the parameters encoding the molecular structure of the peptides and retention. More complex machine learning methods should be applied to successfully capture these complex, often non-linear relationships. The somewhat inconsistent results are also due to the fact that the gradients need to be carefully optimized for a particular separation [13]. On the other hand, the implication of this conclusion is that both QSRRs and our elution order prediction method can assist in localizing optimal conditions in RP-LC method development.

Finally, the developed methodology has great potential for practical applications once it is comprehensively validated on new high-quality retention data of complex mixtures, such as proteomics and metabolomics mixtures. We believe that not only retention time predictions, but also predictions of the elution order have the potential to greatly facilitate peptide/metabolite identification. This can be achieved by comparing classification rates (expressed as false detection rate) before and after implementation of elution order prediction within an RP-LC-MS/MS proteomics or metabolomics workflows and is an integral segment of our future work.

## 3. Materials and Methods

### 3.1. Chromatographic Measurements

Detailed information about measurements of analysis on high-performance liquid chromatography (HPLC) are shown in Table S1 and previous studies [13,17]. Briefly, all the chromatographic measurements were performed with a flow rate of 1 mL/min, and UV detection wavelengths at 214 and 223 nm. The injected sample volume was 20 μL. The mobile phase for case study 1 was methanol and 100 mM tris buffer at pH values of 2.5 and 7.2. For case study 2, gradient elution was carried out with solvent A (water with 0.12% trifluoroacetic acid) and solvent B (acetonitrile with 0.10% trifluoroacetic acid). The mobile phase was filtered through a GF/F glass microfiber filter (Whatman, Maidstone, UK) and subsequently degassed with helium during the analysis. Peptide samples were dissolved in water containing 0.10% (*v*/*v*) of trifluoroacetic acid.

### 3.2. QSRR Model Development

In this work, two QSRR models were developed, for two case studies involving simple analytical mixtures: (i) separation of organic molecules in a Supelcosil LC-18 column, and (ii) separation of peptides in seven columns under varying chromatographic conditions. In the first case study, retention times of 62 organic compounds analyzed in the Supelcosil LC-18 column (Table S1) were utilized. The following retention time prediction model was used:

(1) $$t_{R}=f\left(µ,\;\delta_{\min},\;SASA\right)$$

where *μ* represents the total dipole moment, *δ*_min_ is the electron excess charge of the most negatively-charged atom, whereas *SASA* is the solvent accessible surface area. The model defined with Equation (1) was used because it is one of the earliest, and well-described mechanistic QSRR models derived purely in silico introduced by Kaliszan et al [12,18]. The descriptors *μ* and *δ*_min_ accounted for electrostatics. Namely, dipole-dipole and dipole-induced dipole interactions of the analyte with the mobile and stationary phases; and local polar interactions, respectively. On the other hand, SASA accounted for dispersive interactions of the analyte and the mobile and stationary phase [12].

The dataset itself was obtained from literature [17], while the molecular descriptors were re-calculated using density functional theory (DFT) at a higher level of theory, namely, MN15/6-311+G** [19,20]. Due to a pronounced solvent effect, the implicit SMD solvation model [21] was used to model it. All the DFT calculations were performed in Gaussian 16 software (Gaussian, Inc., Wallingford, CT, USA).

The second case study comprised of retention times of 98 synthetic peptides analysed on seven different columns in varying conditions: Xterra MS C18, LiChrospher RP-18, LiChrospher CN, Discovery HS F5-3, Discovery RPAmide C16, PLRP-S and Chromolith (Table 2 and Table S1). The QSRR formulation used in the second case study is as follows:

(2) $$t_{R}=f\left(\log Sum_{AA},\;\log\nu DW_{vol.},\;\;c\log P\right)$$

where log *Sum_AA_* is logarithm of the sum of gradient retention times of the amino acids composing the peptide, *c*log*P* is the logarithm of its theoretically calculated *n*-octanol−water partition coefficient representing hydrophobicity of the peptides, whereas log *vdW*_vol._ is the logarithm of the peptides’ van der Waals volume. The log *Sum_AA_* descriptor, thereby, accounted for the primary structure of the peptides, whereas the *c*log*P* and *vdW*_vol_ explained most of the remaining variance in retention due to post-translational modification and acetylation [22,23,24] Both the retention data and the molecular descriptors were obtained from literature [13]. In the second case study, due to the sheer size of the molecular structures of most of the analytes, the descriptors were not re-calculated at a higher level of theory.

For a functional form for Equations (1) and (2), a linear equation is employed:

(3) $$t_{R}=\;\alpha_{0}+\alpha_{1}x_{1}+\alpha_{2}x_{2}+\ldots +\alpha_{n}x_{n}+\varepsilon$$

where *x_i_* and *α_i_* denote the molecular descriptors and regression coefficients, respectively, while *ε* is the error. The regression coefficients are typically estimated using multiple linear regression (MLR) [25].

### 3.3. QSRR Model Validation

In QSRR, model validation is the task of demonstrating that the model is a reasonable representation of the actual system; i.e., it reproduces system behavior with enough reliability to satisfy prediction objectives. In this work, both datasets were separated into training and external validation sets using the Kennard and Stone algorithm [26] (70/30% ratio). Performance metrics, such as %RMSE, were evaluated and the predictive ability of the developed models was also depicted.

Chemical domains of applicability were also defined for all the models in both case studies. This was to ensure that the predictions were restricted to prediction of a model to compounds which possessed similar structural, physicochemical, or biological space information similar to the training compounds. For this purpose, the Williams plot, a graphical description of dependence between standardized residuals and leverages of the model was employed. Its warning limits were set as the critical leverage value h* and three multiples of standard deviation. The critical leverage [27] is defined as:

(4) $$h^{*}=\;\frac{3\left(K-1\right)}{N}$$

where *K* is the number of variables, and *N* is the number of observations.

### 3.4. Elution Order Prediction

As previously mentioned, elution order in HPLC is generally governed by polarity. Thereby, in RP-HPLC, solutes will typically be eluted in a reverse order to nonpolar compounds where retention increases with decreasing polarity. However, it is not always as straightforward. In this work, the elution order problem was formulated by considering all the peaks. Mathematically, elution order can be easily predicted once retention time is predicted and then sorted in ascending order. Specifically, indices are defined for all the experimental peaks (sorted in ascending order). Subsequently, retention times predicted by a QSRR model are sorted with respect to the experimental times and another set of indices is defined and sorted. The resulting differences between the two sets of indices define the elution order error. Therefore, it is directly implemented within the QSRR modelling process.

### 3.5. Multi-Objective Optimization (MOO)

Multi-objective optimization (MOO) seeks to optimize a vector-valued cost function, with more than one objective. In this work, the MOO formulation of elution order prediction was defined as:

(5) $$\alpha^{*}=\arg min\left(f_{1}\left(\alpha \right),f_{2}\left(\alpha \right)\right)\;f_{1}\left(\alpha \right)=\%RMSE\left(t_{R}\right);\;f_{2}\left(\alpha \right)=\%RMSE\left(order\right)$$

where α* represent optimal regression coefficients. %RMSE(*t_R_*) and %RMSE(order) denote %RMSE of retention time and that of elution order. Upon obtaining the MLR model (control), the MLR coefficients were used as an initial point for multi-objective optimization (MLR-MOO). In this work MOO is implemented using genetic algorithms (GA) [28,29]. GAs are a family of optimization algorithms based on natural evolution and were chosen as a robust alternative to the classical interior-point [30] or conjugate gradient [31] algorithms.

The solution to the MOO problem is not a single point, but a family of points (Pareto front). Each point on the Pareto front that does not lead to degradation is optimal in the sense that no improvement can be achieved in one vector component of the cost with respect to one of the remaining components. From the obtained Pareto front(s), a solution is selected which gives a desirable trade-off between the objectives, in this case decrease of elution order error at the expense of retention time prediction. It has to be noted that the choice of an upper bound for the loss of the retention predictive ability (expressed as 100 − %RMSE) is a user-defined parameter.

### 3.6. Objective Functions for MOO

For MOO, %RMSE of retention time and elution order were used as objective functions for elution order prediction through MOO. %RMSE(*t*_R_) [15,32] was defined as follows:

(6) $$\%RMSE\left(t_{R}\right)=\sqrt{\frac{\sum_{i=1}^{n}\left(\frac{t_{R,pred.,i}-t_{R,obs.,i}}{t_{R,obs.,i}}\right)}{n}}$$

In Equation (6) *t*_R,pred_ and *t*_R,obs._ stand for the predicted and experimentally-obtained retention times, respectively, while *n* is the number of observations. Once the predicted retention times are sorted in ascending order with respect to the experimental ones, the elution order can be easily predicted. Subsequently, %RMSE(order) had the same form as the equation for %RMSE(*t*_R_) with the experimental and predicted elution order instead of the retention time.

### 3.7. Selection of an Optimal MOO Solution

An optimal solution from the obtained Pareto set of solutions is selected employing the following approach. Starting from the knee point of the Pareto front, a point which represents the optimal trade-off between errors in retention time and elution order is selected. This is because the estimated Pareto front knee point may not always represent an acceptable solution to the end-user. Thereby, the optimal MOO solution can be considered as a user-defined parameter. Subsequently, the optimal solutions are compared to the control QSRR models built via MLR. Diagnostic metrics: (i) predictive performance, and (ii) (analytical chemical) domain of applicability, are used to evaluate the developed QSRR and QSRR-MOO models.

### 3.8. Sum of Ranking Differences

An attempt was made to utilize sum of ranking differences (SRD) [33] for an objective selection of a solution from the Pareto front. However, for all the columns the differences were too subtle. Instead, SRD was used to directly compare the performance of all the RP-LC columns of CS2 in terms of their elution order.

SRD is a method that has been used on several occasions for comparison of chromatographic columns both in terms of column performance and QSRR model performance [34,35]. Essentially, SRD is an unbiased ranking method with the ability to compare models, methods, analytical techniques, and so on. It is based on the sum of squared differences in the ranking of the observed objects (e.g., chromatographic columns) and their respective observations (e.g., analytes). The ranking is obtained with respect to either a golden standard or a global statistical metric (such as mean, or median) of the observations. In this work, an extension of the original SRD method, which includes validation through a comparison of ranks of random numbers (CRNNs) has been employed [36]. SRD-CRNNs are based on computing SRD values for a series of normally-distributed random numbers. Values of the first icosaile, median, and last icosaile are computed, as well as a sufficient number of points to plot the SRD normal distribution curve. If an object has an SRD value on either side of the curve, it is statistically significantly different than the ranking of random numbers.

### 3.9. Software Development

Implementation of both the GA in its multi-objective form, objective function, knee point determination, and comprehensive QSRR model validation was carried out using MATLAB 2019a (MathWorks, Sherborn, MA, USA). A graphical interface was constructed for the purpose of easing user interaction with the developed software.

## 4. Conclusions

In conclusion, an elution order prediction method was developed based on quantitative structure-retention relationships (QSRRs) and multi-objective optimization (MOO) with two objective functions: %RMSE(*t*_R_) and %RMSE(order). Two case studies were evaluated: (i) separation of organic molecules; and (ii) separation of peptides on several columns with varying chromatographic conditions. Results have shown that, when compared to control calculations, in both case studies for most of the columns the same trend is observed. Retention time error increases, while the elution order error decreases. As evident from our proof-of-concept study, the developed method has the potential for application with respect to complex analytical mixtures, such as proteomics mixtures with thousands of peptides. Future work will entail the application of the method to such complex analytical mixtures, utilization of machine learning, and the development of an online QSRR platform.

## 5. Patents

Patent applied for USPTO (app. no. 16/740,243) and KIPO (app. no. 10-2019-0069924).

## Figures and Tables

**Table 1:** Summary of model performances for the first and second case studies.

CS *^a^*	Column Name	Analysis Parameters *^b^*	Model	%RMSE(*t*_R_)	%RMSE(Order)	SRD/%
I	Supelcosil LC-18	*t*_G_ = 10 min *T* = 35 °C	MLR (control)	8.57	59.07	N/A
			MLR-MOO	9.33	42.04	N/A
II	Xterra MS C18	*t*_G_ = 20 min *T* = 40 °C	MLR (control)	11.50	25.01	8.12
			MLR-MOO	12.07	18.05	7.58
II	LiChrospher RP-18	*t*_G_ = 20 min *T* = 40 °C	MLR (control)	13.25	30.28	7.79
			MLR-MOO	13.31	22.23	8.00
II	LiChrospher RP-18	*t*_G_ = 60 min *T* = 40 °C	MLR (control)	25.60	34.11	10.04
			MLR-MOO	26.84	20.50	7.71
II	LiChrospher RP-18	*t*_G_ = 120 min *T* = 40 °C	MLR (control)	42.31	153.00	14.16
			MLR-MOO	47.43	26.82	9.58
II	LiChrospher RP-18	*t*_G_ = 20 min *T* = 60 °C	MLR (control)	18.45	36.12	8.45
			MLR-MOO	18.58	31.09	7.91
II	LiChrospher RP-18	*t*_G_ = 20 min *T* = 80 °C	MLR (control)	18.82	35.25	8.33
			MLR-MOO	18.83	34.10	8.75
II	Licrospher CN	*t*_G_ = 20 min *T* = 40 °C	MLR (control)	39.28	195.82	13.20
			MLR-MOO	40.85	31.08	10.37
II	PLRP-S	*t*_G_ = 20 min *T* = 40 °C	MLR (control)	20.07	69.44	9.95
			MLR-MOO	21.89	44.54	10.41
II	PLRP-S	*t*_G_ = 60 min *T* = 40 °C	MLR (control)	37.92	107.94	13.41
			MLR-MOO	39.28	46.84	9.83
II	PLRP-S	*t*_G_ = 20 min *T* = 60 °C	MLR (control)	21.75	94.97	10.83
			MLR-MOO	21.94	55.19	9.95
II	PLRP-S	*t*_G_ = 60 min *T* = 60 °C	MLR (control)	40.11	321.65	13.24
			MLR-MOO	44.91	47.01	9.54
II	PLRP-S	*t*_G_ = 20 min *T* = 80 °C	MLR (control)	22.36	137.16	12.12
			MLR-MOO	22.71	55.35	10.87
II	PLRP-S	*t*_G_ = 60 min *T* = 80 °C	MLR (control)	42.60	194.56	14.37
			MLR-MOO	45.16	44.38	9.70
II	Discovery RP Amide C16	*t*_G_ = 20 min *T* = 40 °C	MLR (control)	36.73	261.22	17.95
			MLR-MOO	37.58	75.84	15.62
II	Discovery RP Amide C16	*t*_G_ = 20 min *T* = 60 °C	MLR (control)	36.37	219.01	17.62
			MLR-MOO	36.98	70.10	15.66
II	Discovery RP Amide C16	*t*_G_ = 20 min *T* = 80 °C	MLR (control)	36.74	241.63	14.54
			MLR-MOO	38.01	69.92	13.29
II	Discovery HS F5	*t*_G_ = 20 min *T* = 40 °C	MLR (control)	12.81	34.00	10.58
			MLR-MOO	14.43	22.08	9.33
II	Chromolith	*t*_G_ = 20 min *T* = 40 °C	MLR (control)	20.82	43.81	8.20
			MLR-MOO	21.34	32.55	9.25

*^a^ CS—case study. ^b^ t*_G_—gradient retention time.

**Table 2:** Key physicochemical parameters of the evaluated chromatographic columns.

#	Column Name	Length/mm	Internal Diameter (ID)/mm	Particle Size/μm	Carbon Load (C)/%	Pore Size/Å	Surface Area/m^2^ g
1	Xterra MS C18	150	4.6	3.5	15.5	125	175
2	LiChrospher RP-18	250	4.6	5.0	21.0	100	350
3	LiChrospher CN	125	4.6	5.0	6.6	100	350
4	Discovery HS F5-3	150	4.6	3.0	12.0	120	300
5	Discovery RP Amide C16	150	4.6	5.0	11.0	180	200
6	Chromolith	100	4.6	2.0	18.0	130	300
7	PLRP-S	150	4.1	5.0	16.0	100	300
8	Supelcosil LC-18	150	4.6	5.0	11.0	120	170

## Supplementary Materials

The following are available online, Full Gaussian 16 reference (Ref. S1); Table S1. Summary of experimental conditions of the chromatographic analyses for both case studies; Figure S1. Performance of the MLR(control) model; Table S2. Molecular descriptors, experimental retention times and predicted retention times of 62 organic analytes used in the first case study; Table S3. Molecular descriptors, experimental retention times and predicted retention times of 98 synthetic peptides used in the second case study. TableS S2 and S3 are given in form of a spreadsheet in a separate file.
