# Supplementary material for: Prediction of Chromatographic Elution Order of Analytical Mixtures Based on Quantitative Structure-Retention Relationships and Multi-Objective Optimization
Source: Molecules. 2020 Jul 6;25(13):3085. doi: 10.3390/molecules25133085 (PMC7411958; doi:10.3390/molecules25133085)
Supplement: Supplementary file 1 [file molecules-25-03085-s001.zip › 2020-Zuvela_et_al-Supporting_Information-final rev 1.docx]

Supplementary materials

Prediction of Chromatographic Elution Order of Analytical Mixtures Based on Quantitative Structure-Retention Relationships and Multi-Objective Optimization

Petar Žuvela ^1^, J. Jay Liu ^2,^*, Ming Wah Wong ^1^ and Tomasz Bączek ^3^

^1^ Department of Chemistry, National University of Singapore, Singapore 117543, Singapore; petar.zuvela@nus.edu.sg (P.Ž.); chmwmw@nus.edu.sg (M.W.W.)

^2^ Department of Chemical Engineering, Pukyong National University, 48513 Busan, Korea

^3^ Department of Pharmaceutical Chemistry, Medical University of Gdańsk, 80-416 Gdańsk, Poland; tbaczek@gumed.edu.pl

***** Correspondence: jayliu@pknu.ac.kr; Tel.: +82-51-629-6453

Contents:

1. **Reference S1.** Complete Gaussian 16 reference.
2. **Table S1.** Summary of experimental conditions of the chromatographic analyses for both case studies.
3. **Figure S1.** Performance of the MLR(control) in prediction of **A)** retention time, **B)** elution order, and **C)** applicability domain for CS1 (Supelcosil LC, *t*_G_ = 10 min, *T* = 35 °C), **D)** prediction of retention time, **E)** elution order, and **F)** applicability domain for CS2 (Xterra MS C18, *t*_G_ = 20 min, *T* = 40 °C). Blue open circle shapes represent the training set, whereas the pink open diamond shapes represent the testing set observations.

**Ref S1.** Gaussian 16, Revision B.01, Frisch, M. J.; Trucks, G. W.; Schlegel, H. B.; Scuseria, G. E.; Robb, M. A.; Cheeseman, J. R.; Scalmani, G.; Barone, V.; Petersson, G. A.; Nakatsuji, H.; Li, X.; Caricato, M.; Marenich, A. V.; Bloino, J.; Janesko, B. G.; Gomperts, R.; Mennucci, B.; Hratchian, H. P.; Ortiz, J. V.; Izmaylov, A. F.; Sonnenberg, J. L.; Williams-Young, D.; Ding, F.; Lipparini, F.; Egidi, F.; Goings, J.; Peng, B.; Petrone, A.; Henderson, T.; Ranasinghe, D.; Zakrzewski, V. G.; Gao, J.; Rega, N.; Zheng, G.; Liang, W.; Hada, M.; Ehara, M.; Toyota, K.; Fukuda, R.; Hasegawa, J.; Ishida, M.; Nakajima, T.; Honda, Y.; Kitao, O.; Nakai, H.; Vreven, T.; Throssell, K.; Montgomery, J. A., Jr.; Peralta, J. E.; Ogliaro, F.; Bearpark, M. J.; Heyd, J. J.; Brothers, E. N.; Kudin, K. N.; Staroverov, V. N.; Keith, T. A.; Kobayashi, R.; Normand, J.; Raghavachari, K.; Rendell, A. P.; Burant, J. C.; Iyengar, S. S.; Tomasi, J.; Cossi, M.; Millam, J. M.; Klene, M.; Adamo, C.; Cammi, R.; Ochterski, J. W.; Martin, R. L.; Morokuma, K.; Farkas, O.; Foresman, J. B.; Fox, D. J. Gaussian, Inc., Wallingford CT, 2016.

**Table S1.** Detailed information about the manufacturer and high-performance liquid chromatography (HPLC) analysis.

| # | Column name | Manufacturer | Packed with /  made of | Column size / cm | Particle size / μm | Pore size / Å | Surface area / m^2^ g^-1^ | Gradient time / min | Temperature / °C | Gradient / % |
| --- | --- | --- | --- | --- | --- | --- | --- | --- | --- | --- |
| 1 | XTerra MS C18 | Waters, Millford, MA, USA | octadecyl-bonded silica | 15.0 × 0.46 | 3.5 | 125 | 175 | 20 | 40 | 0-60 |
| 2 | LiChrospher RP-18 | Merck, Darmstadt, Germany | octadecyl-bonded silica | 25.0 × 0.46 | 5.0 | 100 | 350 | 20 | 40 | 4-60 |
|  |  |  |  |  |  |  |  |  | 60 |  |
|  |  |  |  |  |  |  |  |  | 80 |  |
|  |  |  |  |  |  |  |  | 60 | 40 |  |
|  |  |  |  |  |  |  |  | 120 | 40 |  |
| 3 | LiChrospher CN | Merck, Darmstadt, Germany | octadecyl-bonded silica terminated with cyano groups | 10.0 × 0.46 | 5.0 | 100 | 350 | 20 | 40 | 4-60 |
| 4 | Discovery HS F5-3 | Supelco, Bellefonte, PA, USA | pentafluorophenyl-propyl- terminated reversed phase | 15.0 × 0.46 | 3.0 | 120 | 300 | 20 | 40 | 4-60 |
| 5 | Discovery RP Amide C16 | Supelco, Bellefonte, PA, USA | silica terminated with amide groups | 15.0 × 0.46 | 5.0 | 180 | 200 | 20 | 40 | 4-60 |
|  |  |  |  |  |  |  |  |  | 60 |  |
|  |  |  |  |  |  |  |  |  | 80 |  |
| 6 | Chromolith | Merck, Darmstadt, Germany | silica highly porous monolithic rod of silica | 10.0 × 0.46 | 2.0 | 130 | 300 | 20 | 40 | 4-60 |
| 7 | PLRP-S | Polymer Laboratories Amherst, MA, USA | cross-linked polystyrene (divinylbenzene) | 15.0 × 0.41 | 5.0 | 100 | 300 | 20 | 40 | 4-60 |
|  |  |  |  |  |  |  |  |  | 60 |  |
|  |  |  |  |  |  |  |  |  | 80 |  |
|  |  |  |  |  |  |  |  | 60 | 40 |  |
|  |  |  |  |  |  |  |  |  | 60 |  |
|  |  |  |  |  |  |  |  |  | 80 |  |
| 8 | Supercosil LC-18 | Supelco, Bellefonte, PA, USA | octadecyl-bonded silica | 15.0 × 0.46 | 5.0 | 120 | 170 | 10 | 35 | 10-90 |

**Table S1.** (cont.)

| # | Column name | Chromatograph | Software | Auto-sampler | Thermo-stat | Pump | Wave-length | Detector |
| --- | --- | --- | --- | --- | --- | --- | --- | --- |
| 1 | Xterra MS C18 | LC Module I plus | Waters Millennium 2.15 | Model Code LCH | Model Code LCH | Model Code LCH | UV-vis | Model Code LCH |
| 2 | LiChrospher RP- 18 | Merck-Hitachi LaChrom HPLC system | D-7000 HPLC System Manager, version 4.1 | L-7200 | L-7360 | D-7000 | UV-vis | L-7400 |
| 3 | LiChrospher CN | Merck-Hitachi LaChrom HPLC system | D-7000 HPLC System Manager, version 4.1 | L-7200 | L-7360 | D-7000 | UV-vis | L-7400 |
| 4 | Discovery HS F5-3 | Not listed | HP Chem Station for LC | HP 1050 | model 7956 | Agilent 1100 | UV-vis | HP 1050 |
| 5 | Discovery RPAmide C16 | Merck-Hitachi LaChrom HPLC system | D-7000 HPLC System Manager, version 4.1 | L-7200 | L-7360 | D-7000 | UV-vis | L-7400 |
| 6 | Chromolith | Not listed | HP Chem Station for LC | HP 1050 | model 7956 | Agilent 1100 | UV-vis | HP 1050 |
| 7 | PLRP-S | Not listed | HP Chem Station for LC | HP 1050 | model 7956 | Agilent 1100 | UV-vis | HP 1050 |
| 8 | Supelcosil LC-18 | Not listed | Waters | Waters Millennium 2.15 | Waters | Waters | Waters | UV-vis |

***References:** Kaliszan, R.; van Straten, M. A.; Markuszewski, M.; Cramers, C. A.; Claessens, H. A. Molecular Mechanism of Retention in Reversed-Phase High-Performance Liquid Chromatography and Classification of Modern Stationary Phases by Using Quantitative Structure–Retention Relationships. *J. Chromatogr. A* **1999**, *855* (2), 455–486. <https://doi.org/10.1016/S0021-9673(99)00742-6>; Ba̧czek, T.; Wiczling, P.; Marszałł, M.; Heyden, Y. Vander; Kaliszan, R. Prediction of Peptide Retention at Different HPLC Conditions from Multiple Linear Regression Models. *J. Proteome Res.* **2005**, 4 (2), 555–563. https://doi.org/10.1021/pr049780r.


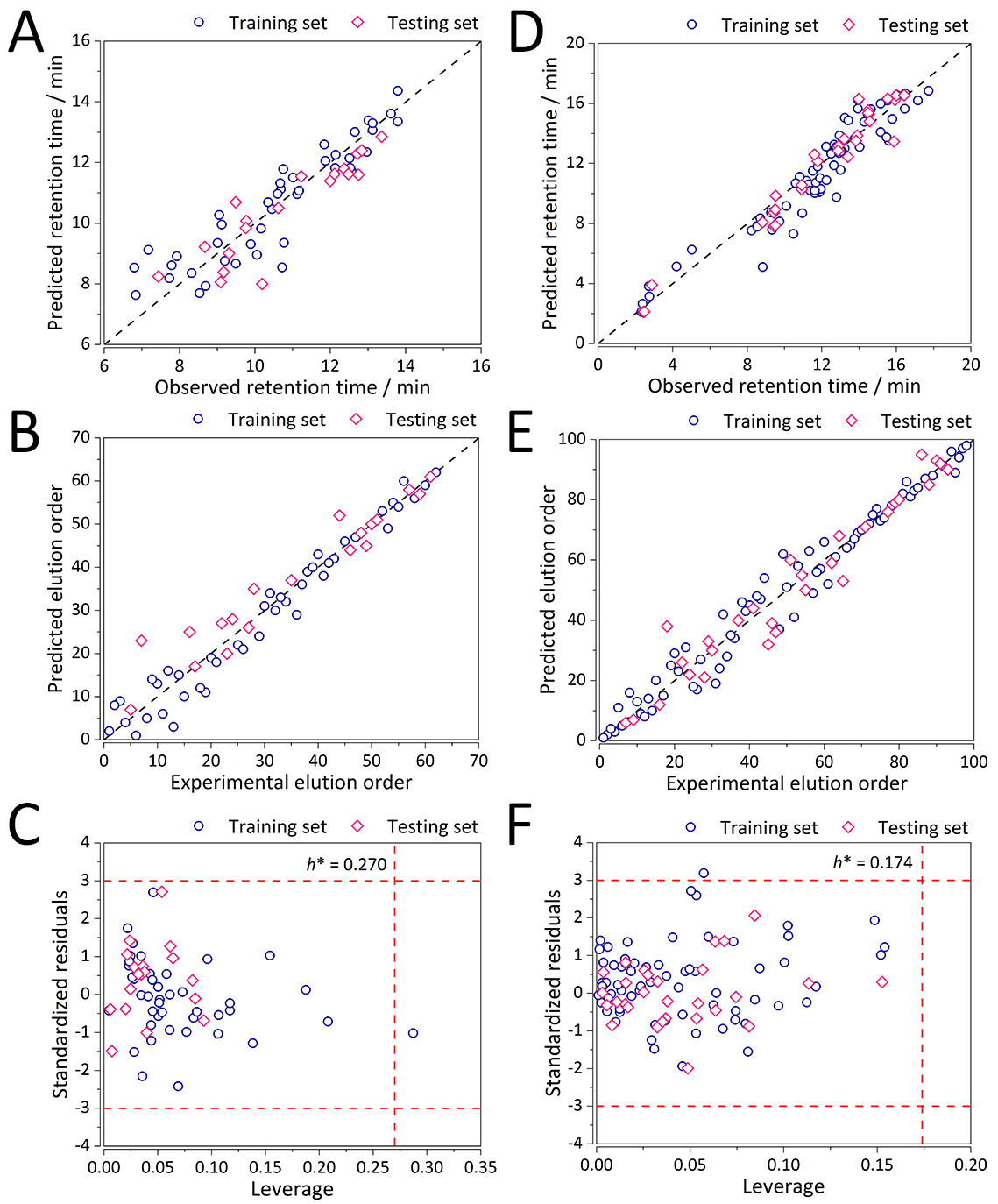


**Figure S1.** Performance of the MLR(control) in prediction of **A)** retention time, **B)** elution order, and **C)** applicability domain for CS1 (Supelcosil LC, *t*_G_ = 10 min, *T* = 35 °C), **D)** prediction of retention time, **E)** elution order, and **F)** applicability domain for CS2 (Xterra MS C18, *t*_G_ = 20 min, *T* = 40 °C). Blue open circle shapes represent the training set, whereas the pink open diamond shapes represent the testing set observations.
